# Signs of developmental stuttering up to age eight and at 12 plus

**DOI:** 10.1016/j.cpr.2006.08.005

**Published:** 2007-04

**Authors:** Peter Howell

**Affiliations:** Department of Psychology and Centre for Human Communications, University College London, Gower St., London WC1E 6BT, England, United Kingdom

**Keywords:** Developmental stuttering, Onset of stuttering, Recovery and persistence of stuttering, Language development

## Abstract

Clinicians who are familiar with the general DSM-IV-TR scheme may want to know how to identify whether a child does, or (equally importantly) does not, stutter and what differences there are in the presenting signs for children of different ages. This article reviews and discusses topics in the research literature that have a bearing on these questions. The review compared language, social–environmental and host factors of children who stutter across two age groups (up to age eight and 12 plus). Dysfluency types mainly involved repetition of one or more whole function words up to age eight whereas at age 12 plus, dysfluency on parts of content words often occurred. Twin studies showed that environmental and host factors were split roughly 30/70 for both ages. Though the disorder is genetically transmitted, the mode of transmission is not known at present. At the earlier age, there were few clearcut socio-environmental influences. There were, however, some suggestions of sensory (high incidence of otitis media with effusion) and motor differences (high proportion of left-handed individuals in the stuttering group relative to norms) compared to control speakers. At age 12 plus, socio-environmental influences (like state anxiety) occurred in the children who persist, but were not evident in the children who recover from the disorder. Brain scans at the older age show some replicable abnormality in the areas connecting motor and sensory areas in speakers who stutter. The topics considered in the discussion return to the question of how to identify whether a child does or does not stutter. The review identifies extra details that might be considered to improve the classification of stuttering (e.g. sensory and motor assessments). Also, some age-dependent factors and processes are identified (such as change in dysfluency type with age). Knowing the distinguishing features of the disorder allows it to be contrasted with other disorders which show superficially similar features. Two or more disorders can co-occur for two reasons: comorbidity, where the child has two identifiable disorders (e.g. a child with Down Syndrome whose speech has been properly assessed and classed as stuttering). Ambiguous classifications, where an individual suffering from one disorder meets the criteria for one or more other disorders. One way DSM-IV-TR deals with the latter is by giving certain classification axes priority over others. The grounds for such superordinacy seem circular as the main role for allowing this appears to be to avoid such ambiguities.

## 1 Introduction

Clinical psychologists (particularly those working in paediatrics) will, as part of their caseload, often encounter clients who they suspect to be stuttering. One reason for this is that stuttering is a sign of other types of communication disorder (e.g. tics, elective mutism) and other classes of mental disorder (e.g. substance related, and anxiety, disorders). A second reason is that stuttering is akin to the normal nonfluency that is observed in all children. One question to decide is whether or not a particular child is stuttering.

It is difficult for a clinician who is not sure about a diagnosis to go on and advise on treatment. In addition, the reported rate of unassisted recovery from the disorder in childhood is high, so it is likely that even in cases of childhood stuttering the problem will resolve of its own accord. A final fact to note is that, as stuttering is similar to normal nonfluency, it might not be considered to be a problem that one needs to be quick to treat. Together these observations can lead general medical practitioners to the decision to “leave stuttering alone and it'll go away” (Yairi & Ambrose, 2005).

The American Psychiatric Association's Diagnostic and Statistical Manual of Mental Disorders Revision IV Text Revision, DSM-IV-TR (APA, 2000), as well as other sources, give advice on stuttering. Some of the issues touched on in DSM-IV-TR are the signs of stuttering, whether speakers who stutter differ in frequency and/or severity of these signs from fluent speakers and speakers with other disorders, and what happens over the period of time that speakers recover or persist from the disorder. Answers to these questions are constantly being debated, researched and revised. The clinician needs to be aware of these deliberations when arriving at a judgment about diagnosis and treatment of stuttering. As a contribution to the debate, recent empirical work about diagnosis of developmental stuttering is reviewed.

The article starts by considering the principal signs of stuttering as described in DSM-IV-TR followed by a section on demographics of the disorder. The review was designed to examine two age ranges, one where the chances of recovery were high and the other where the chances of recovery have been reported to be low or non-existent. Studies which have examined these age ranges were all consulted. The first age range is between onset and eight years (modal onset ages for stuttering are three and five years, Andrews & Harris, 1964). The second is when chance of recovery from stuttering is low (12 plus according to Andrews & Harris, 1964). Environmental and host factors are examined separately at both ages (up to eight and from 12 onwards). The review will serve as basis for a discussion of: 1) diagnostic criteria, 2) specificity of the signs of stuttering (how to decide whether stuttering signs occur in other disorders), 3) comorbidity of stuttering with other disorders, and 4) ambiguity of classifications (risk of classifying stuttering as some other disorder and vice versa).

## 2 Symptomatology and assessment

DSM-IV-TR characterizes stuttering in terms of “frequent repetitions or prolongations of sounds or syllables” (APA, 2000, p.67). The authors make a number of other comments about differential diagnosis, associated features and etiology that are considered later in this review.

It is informative to compare the characteristics listed in DSM-IV-TR with the widely-used set of eight signs associated with stuttering given by Johnson et al. (1959). The signs Johnson et al. (1959) used were: 1) interjections (which includes filled pauses); 2) word repetitions; 3) phrase repetitions; 4) part-word repetitions; 5) prolongations; 6) broken words; 7) incomplete phrases (sometimes called abandonments); and 8) revisions. Johnson frequently pointed out that fluent speakers produced all these types of dysfluency on occasions.

The first six signs of dysfluencies are illustrated in the following two short stretches of speech produced by two children who stutter (both male). The first one was spoken by a child aged five: “one thing er I enjoy is to take my my dog for a walk. Near, near my house there is a canal. Me and my dad set off early in the morning an', and walk along the canal with Tommy, my dog. There's a caff near a bridge where we, where we er sometimes have our, our breakfast.” This child shows interjections (after “one thing” and after “where we”), single word repetitions (“my, my”, “near, near”, “an', and”) and a phrase repetition (“where we, where we”).

The second child was somewhat older (aged 12): “I don't like sssschool. The other kids tease me about my, about my sssstutter. Lessons are OK except when, when I have to speak in class. Th.there are some teachers who p.p.p.pick on me. They make me sp/eak in class mmmore than my, my m.mates, er just to embarrass me.” This child shows an interjection (“er” before “just to embarrass me”) word repetitions (“when, when”, “my, my”) and a phrase repetition (“about my, about my”), signs also seen with the first speaker. Here, in addition, there are part-word repetitions (“th.there”, “p.p.p.pick”, “m.mates”), prolongations (“sssschool”, “ssstutter”, “mmmore”) and a break in a word (“sp/eak”).

Coincidentally, neither of these speakers showed incomplete phrases (non-sequitors) or revisions (change of one utterance by another) in these extracts. There is debate about whether all the remaining signs should be included when assessing whether stuttering is present. Thus Wingate (2001) does not want signs that involve repetition of whole words to be included (categories 2 and 3 on Johnson's list) whereas Yairi, Watkins, Ambrose, and Padden (2001) defend their use. Based on these extracts alone, Wingate would not consider that the first child was stuttering but he would consider that the second child was. Yairi et al. (2001), on the other hand, would consider that both were stuttering. A further feature to note is that signs 4–6, are only observed with the older speaker. Generally speaking, we find that signs 4–6 are more common in older speakers.

## 3 Dysfluency types at age eight and 12 plus

Language characteristics are the main presenting signs, so the issue of what events in speech should be included when indexing severity needs to be resolved. Most authors agree with Yairi and Ambrose (2005) that whole words should be included, for two main reasons. First, dysfluencies involving whole words are considered as providing an important link between normal nonfluency and stuttering (Howell, 2004a; Kolk & Postma, 1997; Pellowski & Conture, 2005). Second, a change from whole- to part-word dysfluencies has been suggested as a sign of persistence (Conture, 1990; Van Riper, 1982). Neither of these changes could be apparent if whole words were excluded from dysfluency assessments.

Of course, these arguments for distinguishing whole- and part-word dysfluencies need to be backed up with empirical support. Before some supporting data are provided, it is necessary to appreciate some features of Wingate's wider perspective about stuttering. He considers: 1) stuttering starts in its persistent form, or as he puts it “Stuttering does not ‘develop’” (Wingate, 2002, p.372); 2) he states that there is “no known cure” (Wingate, 2002, p.11), so in addition to being born with it, you cannot get rid of it; and 3) stuttering should be characterized by part-word dysfluency and pauses, not whole word dysfluencies (Wingate, 2001).

There are two challenges to Wingate's view that can be tested: 1) if stuttering develops, speakers not originally showing part-word dysfluencies could acquire them; 2) if stuttering can be cured (assisted or non-assisted recovery), then some speakers who have part-word dysfluencies should lose them. These predictions have not been tested to date, but can be checked using one of our data sets (Howell & Huckvale, 2004).

The chosen data set is from 76 children who were followed up and who were aged between eight and 12 plus (similar in age profile to participants used by Fritzell, 1976). All the children were diagnosed as stuttering at the outset of the study (around age eight) based on clinical assessments and a measure of stuttering severity (Riley, 1994, Stuttering Severity Instrument-3, SSI-3). The children were assessed by SSI-3, by parents, by the children themselves and a by researcher as persisting in, or recovered from, stuttering at age 12 plus, at which age stuttering should have recovered if it was going to (Howell, Davis and Williams, 2006, give full details of how the assessments were made). Using these criteria, 54% recovered, which was close to the 47% Fritzell (1976) reported. Though it is not important for these analyses because stuttering cannot be cured according to Wingate, care was taken to ensure that the recordings were obtained outside periods where the children could have been responding to treatment they received, using Finn's (1998) guideline. The data were not collected from stuttering onset, but again this should not matter as, according to Wingate, stuttering does not develop. Thus, the 46% who persisted in their stuttering at the end of the study should also have stuttered at the start and no new speakers should join this group. The data used to test the two predictions consisted of spontaneous speech samples which have been phonetically transcribed both when the children were first recruited and also at age 12 plus. Johnson et al.'s (1959) eight different dysfluency signs were coded on each speech sample and counts of dysfluency signs 2–3 and 4–6 on two-minute extracts were made, participant by participant.

Using number of dysfluencies in categories 4–6, the data were cast into a two-by-two contingency table where one contingency was whether the speaker was in the bottom or top 50% at outset and the other was whether they were in the bottom or top 50% at the end of the study. If speakers maintain their stuttering pattern, they should either lie in the bottom 50% at outset and end (top left entry in Table 1; not stuttering according to Wingate) or be in the top 50% at outset and end (bottom right entry in Table 1; stuttering according to Wingate). Clearly there are many cases that fall outside these cells. In particular speakers who were originally in the bottom 50 percentile and move to the top 50 percentile (bottom left) have acquired stuttering signs, and speakers who were originally in the top 50 percentile and move to the bottom 50 percentile (top right) have reduced the signs of stuttering that Wingate (2001) deemed true indications of the disorder. Thus, both predictions that challenge Wingate's view (that stuttering can be acquired, top right cell, and that stuttering can recover, bottom left cell) were upheld based on the signs he singled out (4–6). More speakers seem to recover (16) from these signs than acquire them (11).

Essentially the same happens if the more rigorous criteria for designating the participants as persistent or recovered at age 12 were used (SSI-3, parent, child and researcher scores). Table 2 shows the counts of children initially in the top or bottom 50 percentile of counts of signs 4–6 at outset (rows) against designation at 12 plus as recovered or persistent (columns). The thing to note, once again, is that there are appreciable numbers of participants in the top right (acquired signs 4–6) and bottom left (lost signs 4–6) which should not occur according to Wingate (2001).

To summarize this section, part-word stuttering (signs 4–6) can be acquired or lost over development.

## 4 Demographics

Incidence and point prevalence were reported by Andrews and Harris (1964). They studied stuttering in 1142 families in Newcastle-on-Tyne with children born between May and June 1947. The study (usually referred to as the 1000-Family Survey) ended in 1962 when the children were aged 15, at which time 750 children were still participating. The authors established that, for the children in the 1000-Family Survey, the point prevalence of stuttering was about 1%. Incidence up to age 15 was about 5%. Most cases started between ages three and five years. More boys stuttered than girls (overall a ratio of 2.4:1) and the ratio increased as the children got older (indicating that girls recovered from stuttering at an earlier age than boys).

A problem in studies of lifetime prevalence with disorders that are infrequent (as is stuttering) is that a large sample is needed in order to provide a reasonable sized (and statistically reliable) sub-sample with the disorder. When a large sample has to be examined, it is difficult to assess each individual in depth. In the 1000-Family Survey, the children were seen first by health visitors. Given that even experts have difficulty in assessing stuttering (Kully & Boberg, 1988), the screening by the health visitors is likely to have missed some cases and misdiagnosed others. The misdiagnoses as stuttering would have been picked up as all cases revealed after the initial screening were examined by speech-language pathologists. However, this does not apply to the missed cases. Also, the estimates of incidence and prevalence have been questioned (Ingham, 1976) because of the 33.2% attrition rate over the course of the study.

Given these problems, the expense involved in such studies and the increased rate of mobility of families that would further exacerbate maintaining contact, nothing comparable in terms of size of the sample employed, duration over which the children were followed up and range of factors investigated has been attempted since the 1000-Family Survey. The closest thing is a study by Mannson (2000) which examined 1040 children born on a Danish island over a two year period (98% of all the births in this period). The island has a low-mobility population of about 45,000 individuals. The author and a team of four clinicians assessed speech, hearing and language in face-to-face interviews. Incidence of stuttering was estimated at 4.9% of the children after their third birthday and 5.09% after two follow-ups several years later. The incidence estimates over these periods are close to the estimates reported in Andrews and Harris (1964).

There are also some recent longitudinal data from the Twins Early Development Study (TEDS) (Trouton, Spinath & Plomin, 2002). TEDS is a database of all twins born between 1994 and 1996 in the United Kingdom. Data are available about stuttering when the twins were aged two, three, four and seven. Dworzynski, Remington, Rijksdijk, Howell and Plomin (in press) reported that the point prevalence of stuttering ranged between 1–3% for the different ages, with most cases reported at age four. It was 1.1% (118 children), 2.0% (227 children), 3.5% (493 children) and 1.6% (218 children) at ages two, three, four and seven respectively. Incidence across all the test ages was 1812 (7%), with 1029 boys (4%) and 783 girls (3%), who had been rated as stuttering out of all of the twins (*N* = 25,830) in the entire data set who had been contacted over the seven-year period. The sex ratio trend increased slightly with age: for each girl who stuttered at ages two and three, there were approximately 1.6 boys, whereas at ages four and seven there were approximately 1.8 boys for every girl who stuttered (same value for the two ages).

Apart from Andrews and Harris (1964), Dworzynski et al. (in press), and Mannson (2000), there have been few other longitudinal studies that have used an unselected target sample of fluent speakers and speakers who stutter. Kloth, Kraimaat, Janssen, and Brutten (1999) used a selected sample which followed 93 high risk children (born into families with a stuttering parent). Two years after the project commenced, 23 children were reported to stutter, and four years after that 70% of the children who had stuttered at the start of the project had recovered. There have been a number of reports which specifically targeted samples of speakers who stutter. Ryan (2001) followed up 22 two- to three-year-old children for two years and reported a 68% recovery rate. Rommel, Hage, Kalehne, and Johannsen (2000) reported follow-up results for 65 five-year-old children three years into their study and reported a 71% recovery rate. The largest scale investigation that has followed up children who stutter is an on-going study by Yairi and Ambrose (2005). They have confirmed Andrews and Harris's (1964) demographic findings (sex ratio and the high rate of spontaneous recovery). They reported that most cases of stuttering have their onset at 24–36 months.

Other ways in which information about persistence and recovery rates have been obtained have employed less robust methods for collecting data. The principal alternative methods are: a) informal report of cases observed in clinics over a period of time, and b) retrospective studies which involve speakers who are fluent self-reporting that they had a stutter in the past. Estimates of recovery rates based on clinical observations are somewhat dated and provide values that appear low relative to Andrews and Harris's (1964) survey (e.g. 40% recovery by age eight reported by Bryngelson, 1938). The retrospective studies provide estimates that are closer to those of Andrews and Harris (1964). Thus, Sheehan and Martyn (1966) used self reports from adults and reported a recovery rate of 80%.

Andrews, Craig, Feyer, Hoddinott, Howie, and Neilson (1983) conducted an analysis of recovery rates reported in several studies. They estimated that 75% of those stuttering at age four years, 50% of those stuttering at age six years, and 25% of those stuttering at age 10 years, recovered by the time they reached 16 years of age. If the problem continued into teenage, the chance of recovery decreased. This is supported by the Andrews and Harris (1964) 1000-Family Survey where no child who was stuttering after 12 years of age recovered by age 16 years.

One important fact to consider is the range of ages which need to be assessed. As clinicians are interested in whether and how the disorder develops, examination should ideally start at or before onset and continue through to ages where the likely prognosis is determined. (Andrews et al.'s, 1983, observations discussed earlier suggest age 12 would be appropriate for this.) The above review shows that only Andrews and Harris (1964) follow children up from onset to teenage and that most other studies follow children from onset to around eight years. However, there is one study, mentioned earlier, that followed children from age seven–nine through to teenage: the Fritzell (1976) study reported a recovery rate of 47% between seven-years and teenage. This is lower than the estimates of around 80% obtained from the studies conducted near to onset of the disorder that were reviewed earlier. This result is as would be expected since Andrews et al. (1982) showed that most recovery happens when the children are young. If it is not practicable to follow-up children from around two years to age 12 and upwards, then studies like Fritzell's that extrapolate results to the top end of the age range over which recovery can occur are particularly important. Though the Fritzell study cannot provide an absolute estimate of recovery rate (Yairi & Ambrose, 2005), it does indicate the need to examine recovery and persistence between the ages of 7–12 where around 50% of cases recover (as already mentioned, eight is the age at which most longitudinal studies stop).

In summary, three studies suggest a point prevalence of around 1% and estimate incidence of around 5%. The difference between the prevalence and incidence estimates indicates a recovery rate of about 80% in the period from onset to teenage. This recovery rate is confirmed in recent longitudinal work with children who stutter. Recovery rate between ages seven and teenage is about 50%. Most cases of stuttering have an onset in early childhood (around age three years). Studies consistently report that more boys are affected than girls.

## 5 Etiology

### 5.1 Signs of early stuttering

The goal is to provide an indication about what language, environmental and host factors are associated with risk of stuttering. The factors associated with early stuttering will also permit this disorder to be contrasted with later stuttering and other disorders.

#### 5.1.1 Language characteristics up to age eight

Language characteristics are singled out for discussion in a separate section (though they could be included as host-performance variables) as they are both the presenting sign and offer the most immediate indication about progress of the disorder. The section on symptomatology indicates that dysfluencies on whole word (singly or more than one) predominate up to age eight. In addition, the types of word these events occur on are predominantly function in type (Au-Yeung, Howell & Pilgrim, 1998; Au-Yeung, Vallejo Gomez & Howell, 2003; Bloodstein & Gantwerk, 1967; Bloodstein & Grossman, 1981; Dworzynski, Howell, Au-Yeung & Rommel, 2004; Howell, 2004b; Howell, Au-Yeung & Sackin, 1999). Function words are a closed set of words consisting of pronouns, conjunctions, quantifiers and prepositions, and they complement the content word class, which is an open set, consisting of nouns, verbs, adjectives and adverbs. Function words tend to have simpler phonetic characteristics than content words (Dworzynski & Howell, 2004; Howell, Au-Yeung, Yaruss, & Eldridge, in press). The number of occurrences of dysfluency types 2–3 on function words are shown for the recovered (top section) and persistent speakers (bottom section) at outset in the left-most columns of Table 3 (averages of 3.08 and 2.89 per two-minute period). It is of note that these are comparable in rate for the two groups of speakers. In comparison, number of occurrences of dysfluency types 4–6 on content words (second column) is much lower (averages of 1.17 and 1.38 per two-minute period). Again, it is notable that these rates are comparable for both groups of speakers. (The remaining data in Table 3 is discussed further in Section 5.2.1.). By definition, the absolute rates of stuttering on function and content words differ between speakers who stutter and controls. A less obvious fact, is that the ratio of function to content words is similar for speakers who stutter and controls before age 12 (Howell, Au-Yeng, & Sackin, 1999).

Why should young speakers who stutter and fluent children have problems on the simpler (function) word class? Throneburg, Yairi, and Paden (1994) reported one finding that may explain this. They used preschool children who stutter who, according to Table 3, would predominantly show dysfluencies involving whole function words. They examined the word that followed the word with a dysfluency and, in the majority of cases, this was phonetically more difficult than the dysfluent word itself. To be specific, the word following a dysfluency was more likely to have consonant strings, more than one syllable, contain consonants that emerge late in development, or any combination of these than when the preceding word was fluent. From this it appears that dysfluency on the prior word may be in anticipation of the up-coming difficulty (Clark & Clark, 1977; Howell, 2004a). Speakers may pause before, or repeat simple words that precede more complex words to stall their attempt at the difficult word.

A high frequency of whole word dysfluencies (Johnson's signs 2–3) is a sign of early stuttering, and the chances of recovery when speakers show this pattern are high. This type of dysfluency may arise in anticipation of difficult words that are coming up.

#### 5.1.2 Estimate of environmental influence (twin studies)

A gross (and not absolute) distinction is drawn between social/environmental and host factors. An estimate of environmental effects can be obtained by partialing out genetic effects using incidence rate of stuttering in monozygotic (who share all genetic material) and dizygotic (who share 50% of genetic material) twins. The results of four such studies have been reported (Andrews, Morris-Yates, Howie, & Martin, 1991; Dworzynski et al., in press; Felsenfeld et al., 2000; Ooki, 2005). The studies all show similar figures of 15–30% attributable to the individual's unique environment. Specific environmental factors which could make up the 15–30% contribution are considered next.

##### 5.1.2.1 Social and environmental variables

The social and environmental variables are treated together; social influences arise out of interactions with others in the environment.

###### 5.1.2.1.1 Home environment

In their monograph, Andrews and Harris (1964) reported the results of a second study which documented the influence of several environmental factors that might predispose a child to stutter. The study employed the data of 80 children aged 10–11 + who were identified as stutterers by their head teacher. An equal number of fluent controls from the class of each child who stuttered was selected. The children were older than eight, but the data that predate information specific to the date of the survey, such as home environment, are considered here. They found children who stutter came from the same socio-economic classes as controls. Analysis of the 76 persistent and recovered children who stutter whose speech data were reported in the symptomatology section, confirmed the related finding that there is no association between occupation of the primary wage earner (manual versus non-manual) and persistent/recovered.

Andrews and Harris's study also showed that the mothers of their 80 children who stuttered had a significantly poorer work and school record than those of the control children. The last factor may predispose a child to stutter, though its influence should not be exaggerated as social mores in all sectors of society have changed dramatically since the study was performed.

###### 5.1.2.1.2 Language development

Andrews and Harris (1964) found that the 80 children who stuttered tended to be late in starting to talk (about four months late in acquiring speech) compared to controls. Five of the children in our survey of 76 (6.6%) were reported to be late talkers (two turned out later to be persistent and three recovered). Andrews and Harris's stuttering children also had a significantly higher reported frequency of history of abnormal articulation compared to the controls.

In contrast, Yairi and Ambrose (2005) reported that there were no pervasive expressive language difficulties in their young children who stutter (p.241). In our study on 76 older children in this age range, fourteen (18.4%) had other language disorders (six persistent, eight recovered). A high rate of comorbidity with other language disorders has been reported by Arndt and Healey (2001) which may explain this. Alternatively, the high comorbidity rate might be a reflection of parents over-reporting stuttering and other language disorders (wittingly or unwittingly) because of concern about their child's welfare.

An additional factor examined in our study of 76 children was whether a language other than English was spoken in the home (one requirement for inclusion in our study of 76 children was that the children's first language was English). Thirteen (17.1%) families also spoke a second language. Though all these language factors have been treated here as socio-environmental; in the case of late talking and comorbidity, they could also be host factors. The factors point to a tentative conclusion that a poor language environment may contribute to a small extent to stuttering around age eight.

###### 5.1.2.1.3 Intelligence

The Andrews and Harris study reported that the 80 stuttering children had lower Wechsler IQ scores than controls, and this applied to verbal and non-verbal sub-tests. Overall Wechsler score results showed 31/80 stuttering children had IQ scores below 90 compared with 13/90 controls. Other empirical evidence also supports the view that children who stutter score significantly lower on intelligence tests than do fluent controls. Thus, studies with school-age children who stutter have shown deficits which occur in both verbal and non-verbal intelligence tests (Okasha, Bishry, Kamel, & Hassan, 1974; Schindler, 1955). As non-verbal intelligence is affected, it appears unlikely that the IQ deficits could be explained by difficulties in communication because of stuttering. Though these studies are dated, they suggest that intelligence affects whether a child starts to stutter. In contrast, the recent report by Yairi and Ambrose (2005) indicates that the children in their study scored 13 (persistent) to 22 (recovered) IQ points higher than norms on the non-verbal Arthur adaptation of the Leiter scale.

It has also been reported that the intelligence and social class of children who stutter and are receiving treatment is above average (Cox, 1982). This could be because intelligence and social class contribute, in part, to access to health care. Overall, though there are some suggestions in the early literature that there is a disproportionate number of children who stutter around one standard deviation below the mean compared to controls (Andrews & Harris, 1964). This effect is contradicted by more recent work and by contemporary work on children in therapy.

###### 5.1.2.1.4 Anxiety

Andrews and Harris (1964) used Sarason, Davidson, Lighthall, Waite, and Ruebush's (1960) General Anxiety Scale for Children to assess anxiety. No difference was reported in anxiety at interview between the 80 children who stuttered and their controls. Similar results were obtained by Yairi and Ambrose (2005) using two scales (child anxiety and revised children's manifest anxiety scale). Neither showed any difference, either between persistent and recovered speakers or either of these groups relative to controls.

###### 5.1.2.1.5 Temperament

It has been suggested that a child with a vulnerable or sensitive temperament may be prone to developing, and continuing, stuttering (e.g., Conture, 2001; Guitar, 1998; Zebrowski & Conture, 1998). A child who stutters may have a different temperament to fluent speakers whether or not the temperament variables have anything to do with the fluency problem. Therefore, the first question to ask is whether there are any differences in temperament between children who stutter and controls. There are three studies on this, which all used the children's behavior style questionnaire (BSQ, McDevitt & Carey, 1978), a questionnaire that is filled in by parents. Embrechts, Ebben, Franke, and van de Poel (2000) found 3–7;7 year-old children who stutter were judged to have reduced attention span and less success in adapting to new environments relative to controls. Anderson, Pellowski, Conture, and Kelly (2003) found that 3–5;4 children who stutter were significantly less likely to adapt to change, were less distractible and displayed greater irregularity with biological functions. Howell et al. (2004), found four dimensions differed significantly between 3;7–7;2 children who stutter and controls. As was the case for both the Anderson et al. and Embrechts et al. studies, children who stutter were found to be non-adaptable. In addition, Howell et al. (2004) found that children who stutter were significantly more active, more negative in mood, and less persistent.

There is one finding that is consistent over the Anderson et al. (2003), Embrechts et al. (2000) and Howell et al. (2004) studies. All three reported that children who stutter were less adaptable than their controls. However, none of the remaining temperament dimensions matched in significance and direction across the studies. The first two studies found differences between children who stutter and controls in terms of distractability. Here, however, the direction differed between the two studies (Embrechts et al. found children who stuttered were more distractible than the controls whereas Anderson et al. (2003), found the opposite). Howell et al. (2004) found that children who stuttered were significantly more active, more negative in mood, and less persistent, but none of these dimensions were significant in the other studies.

One study has examined whether temperamental factors are related to fluency problems (Howell et al., 2004). These authors measured temperament variables from BSQ and correlated them with a number of language measures in a group of fluent three-year-olds. Temperament measures did not correlate significantly with: a) a measure of vocabulary (the Oxford version of the communication development inventory (Hamilton, Plunkett, & Schafer, 2000), b) a test that required the child to name pictures they know that uses number of errors in naming the pictures as the performance measure (based on Gershkoff-Stowe & Smith, 1997), c) mean length of utterance, which is a standard measure for the development of productive syntax at these ages (Brown, 1973), d) a measure of the child's ability to understand syntactic relations, which is a perceptual test (the reception of syntax test, Howell, Davis, & Au-Yeung, 2003). Based on this single study, it appears that temperament does not affect fluency and vice versa.

Though, intuitively, temperamental variables would appear to impact on speech-language development and fluency in particular, the evidence at present is not strong. Thus, there is a lack of consistency between which temperamental variables differ between speakers who stutter and controls, and the direction of differences reported across the two groups of speakers do not correspond. Also, the lack of correlation between temperament and language measures suggests that the former is neither influenced by, nor has a direct influence on, language. The lack of consistency between studies on temperament may be a reflection of the heterogeneity of the disorder. The lack of a specific link to fluency raises the possibility that treating temperamental factors per se may not have an impact on fluency. The latter may apply to some of the other variables considered above (e.g. anxiety) where the relationship between this and stuttering has not been examined in early childhood.

###### 5.1.2.1.6 Attitude to stuttering and bullying

Some brief mention also needs to be made of studies into the child's awareness of and attitude to their stutter, and whether the child who stutters is bullied. In the first studies, researchers investigated the attitudes of adults to their stutter. They generated sets of questions intended to establish how adults felt about their speech in different situations. Recently, communication attitude tests have been developed for work with English children, form CAT (Brutten, 1985), and Dutch children, form CAT-D (Vanryckeghem & Brutten, 1992). Both CAT (Boutsen & Brutten, 1989) and CAT-D (De Nil & Brutten, 1991) showed children who stutter had poorer attitudes to their speech than children who do not stutter The internal, and test–retest, reliability of the CAT and CAT-D have been demonstrated in studies (Brutten & Dunham, 1989; Vanryckeghem & Brutten, 1992). Vanryckeghem (1995) considered that the scope of the CAT is limited in that it requires a child to have the ability to read and understand the concepts covered by the test items and consequently it is not generally accurate when used with children younger than 7 years of age. Work is taking place by Vanryckeghem to develop a verbal form of CAT for use with preschool age children.

Vanryckeghem (1995) maintained that the CAT and CAT-D are useful clinical and research tools for evaluating between-group differences when investigating communication attitudes. Yairi and Ambrose's (2005) study showed children acquire awareness of the problem at an early age. Attitude and awareness is not going to occur until a child has contracted the disorder. It may conceivably accelerate progress of the disorder through to age eight, though there is no evidence for, or against, this at present.

One might also speculate that investigations into awareness of stuttering in recovered speakers who used to stutter are less important than investigations into ways of training recovered speakers to be aware of their fluency. On the one hand, awareness of stuttering in recovered speakers appears to be pervasive, as authors who were stutterers often stress the fact that their fear of stuttering never recedes. However, this ‘fear of stuttering’ is likely to be the same feeling that fluent speaker experience in situations like speaking in public. It could easily be the case that if speakers who stutter regard this normal feeling as a hangover of their stuttering, it could be a barrier to recovery. The question then is how to make recovered stutterers appreciate that everyone has this experience rather than to reinforce the view that stuttering never goes away.

Retrospective studies with adults show that they report having been bullied at school. Recently, sociometric techniques have been used which show that children who stutter are likely to be victims of bullying (Davis, Howell, & Cook, 2002). Here, as with awareness, it is unlikely that bullying predated stuttering. Also, there is no evidence showing that children started stuttering because they were bullied, though it is not inconceivable that this might occur. Bullying may, as with awareness, accelerate the progress of the disorder or whether speakers recover or persist, but again there is no evidence for these.

###### 5.1.2.1.7 Summary on socio-environmental factors on young speakers

Up to 30% of variance in liability to stuttering appears to be environmentally determined. In older, but not more recent studies, the home environment, the language community the child is brought up in and IQ appear to have some influences. The evidence for involvement of IQ in the older studies, in particular, seems consistent. There is limited information about whether anxiety affects stuttering in young children, though what studies there are suggest levels of anxiety in young children who stutter are no higher than in controls. Temperament also appears to differ between children who stutter and fluent controls, though its sub-components do not differ between the two groups in any consistent way across the different studies. Temperament does not appear to correlate with stuttering (the same may apply to anxiety at these ages, though this is not known at present). The importance to the clinician of studies that examine direct influences on fluency has been highlighted. Finally, attitude to, and awareness of stuttering and whether a child is bullied are problems that arise after the child has the disorder and, at present, the role these variables could have in the subsequent pace at which the disorder progresses is not known. These issues would appear to merit future investigation. Based on the research findings reviewed, clinicians should not be over reliant on any of the environmental factors that may impact on stuttering. That said, they should be alert to potential problems caused by a poor home environment, the language community in the child's circle of family and friends, low IQ and the child's temperamental ability, particularly as regards to dealing with communication demands.

#### 5.1.3 Host factors up to age eight

In this section, the question considered is whether individuals who stutter at age eight are limited in some way by their performance ability or by their biological makeup.

##### 5.1.3.1 Performance

###### 5.1.3.1.1 Sensory

The DSM-IV-TR states that “Speech difficulties may be associated with a hearing impairment or other sensory deficit or a speech motor deficit.” (APA, 2000, p.68). In line with these expectations, work has examined sensory and motor performance on the 76 children we have followed up. In this section, results are reported for information obtained on the children at the youngest age they were seen. Our initial work asked for self reports of whether the child had had hearing problems at or before onset of the disorder. More recently, formal audiometric screening, detailed information about childhood hearing disorders and psychoacoustic assessments have been made.

Standard audiograms all indicated that the children we have seen so far had hearing within normal limits. Thus, for these children, gross hearing problems do not seem to be a major factor implicated in stuttering.

A questionnaire was used for assessing general health, which included questions about childhood hearing (Landgraf, Abetz, & Ware, 1999). This revealed a high proportion of children who had otitis media with effusion (OME) (other aspects of general health were at, or above, average for the children who stuttered). Information on OME was available for 63 out of the 76 participants. The incidence of OME was 22.2% overall (14/63). This is higher than in the population at large (Zielhuis, Rach, van den Bosch, & van den Broek, 1990). OME can lead to central hearing problem (Stephenson & Haggard, 1992), which could be a risk factor for stuttering.

It has been proposed that the difficulties associated with other language disorders such as specific language impairment (SLI) stem from problems with processing the temporal structure of sounds (Tallal & Piercy, 1973). Work to test the temporal processing deficit hypothesis has used backward masking stimuli where a masking sound is presented before the probe tone that is to be detected (Wright et al., 1997). Wright et al. (1997) reported higher backward masking thresholds (more masking energy needed to prevent participants reporting presence of the tone reliably), but similar simultaneous masking thresholds, compared with control children. Backward masking deficits with SLI children have proved difficult to replicate, possibly because performance is more variable in disordered populations (Hill, Hogben, & Bishop, 2005). Similar problems of replicability occur in stuttering; one study found reliable differences between children who stutter and controls (Howell, Rosen, Hannigan, & Rustin, 2000) and one did not (Howell & Williams, 2004).

###### 5.1.3.1.2 Motor

Handedness was assessed in our 76 children as this may prove informative about potential laterality differences in motor processing in the brain between children who recover and those who persist. The participants were asked which hand they used to write, which has been reported to give a reliable indication of handedness (McManus, 2002). As has often been reported in the past, there was a high proportion of left-handed children who stutter overall (19.7% were left-handed compared with 10% in the population at large McManus, 2002).

##### 5.1.3.2 Biological

###### 5.1.3.2.1 CNS

Organic substrates of sensory and motor deficits may be revealed by scans and any functional problems may be evident in functional imaging studies. At present there are no such studies with children who stutter before age eight, although recently there has been a large number of reports using various scanning techniques that show CNS differences between people who stutter and fluent controls (the Journal of Fluency Disorders, for example, devoted a special issue to this topic in 2003, volume 28, number 4). These reports have renewed investigation into whether brain injury occurs in children, which could precipitate onset of the disorder. Alm (2005), for instance, stated that 40% of his sample of speakers who stutter reported that they had head traumas. Alm argues these could have led to brain stem damage which could have precipitated the onset of stuttering. This proposal is too recent for it to be evaluated.

Although, as time progresses, scans will be made with younger speakers (our group has one planned), no studies have been reported yet. Scans will need to be made as close to stuttering onset as possible in order to establish whether the problem is structural (in which case it should be present at or before onset) or acquired through functional activity associated with experience of the dysfluency (in which case it would present later).

###### 5.1.3.2.2 Genetics

The corollary to the finding of the twin studies presented earlier, that 15–30% of variance in liability to stuttering is environmentally determined, is that 70–85% is genetic. To identify how genetic transmission occurs, family history was obtained in a third study reported in Andrews and Harris (1964). This found that the risk of stuttering was highest in the male relatives of female stutterers. Family history has also been confirmed as a predisposing factor in Yairi and Ambrose's (2005) Illinois study. As part of their work, Ambrose, Yairi, and Cox (1993) carried out detailed analyses to establish patterns of inheritance, and the form of genetic model that best accounts for their data. They concluded that at least one major gene (major locus) is involved at stuttering onset and that the corresponding inheritance pattern conforms to Mendelian transmission. Other research groups examining family history have found that alternative models fit their data better. For example, one group also found a single major locus component, but they concluded that the inheritance pattern did not conform to inheritance patterns based on Mendelian expectations (Cox, Kramer, & Kidd, 1984).

###### 5.1.3.2.3 Summary

As summarized earlier, it was difficult to pinpoint what factors give rise to the 15–30% environmental contribution. Things seem no more clearcut in the case of host factors at these early ages. However, there are several factors that point to hearing being involved (incidence of OME and one study that reports sensory problems in young children who stutter), although the exact role this has on fluency is unclear. Motor factors also seem to be involved if the high rate of left-handers in the case of children who stutter is a reflection of brain-motor involvement. Although it is possible that there are CNS differences between children who stutter and fluent controls, there is no compelling biochemical or scanning evidence to support this at present. Undoubtedly, in the future technological advances will allow children to be scanned, and such evidence will be provided. Language (proportionately higher incidence of all signs of dysfluency in children referred as stuttering compared to controls) and genetic factors (family history of the disorder) seem the most clearcut indications at these ages. The next question to consider is how this picture changes as children grow older and progress to the period where recovery is less likely.

### 5.2 Signs of stuttering at 12 plus

The 5% point prevalence and the 1% annual incidence rate noted in the 1000-Family Survey (Andrews & Harris, 1964) show four out of five (80%) of the respondents recovered from stuttering and only 20% persisted. A similar recovery rate has been reported in independent studies (Dworzynski et al., in press; Mannson, 2000).

Persistent and recovered cases could differ in two ways: a) persistent speakers may respond in a different way from recovered ones and fluent speakers (persistent speakers *diverge* from fluent speakers); and b) the recovered speakers can change from being like the persistent speakers at an early point in time during the course of the disorder but change to being like fluent speakers subsequently (recovered speakers *converge* on fluent speakers).

#### 5.2.1 Language characteristics at 12 plus

Children who stutter at age 12 plus change the balance between types of dysfluency in different ways depending whether they persist or recover. The data for the recovered speakers (top part of Table 3) show that the average number of dysfluency types 2–3 on function words per two-minute period go down from 3.08 to 1.43 and number of 4–6 on content words goes down from 1.17 to 0.44. The reduction of number of number of dysfluencies in classes 2–3 and of 4–6, represents a proportional reduction of both these to levels shown by fluent speakers (recovered speakers converge on fluent speakers).

The data for the persistent speakers (bottom part of Table 3) show that the average number of dysfluency types 2–3 per two-minute period on function words go down from 2.89 to 1.58 but number of dysfluency types 4–6 on content words goes up from 1.38 to 1.68. The increase in 4–6 on content words, specifically for persistent speakers, shows these speakers diverge from the speakers who recover. One interpretation of the increase of dysfluency types 4–6 on content words is that speakers cease delaying by repeating function words that precede the content word (as observed at age eight) and attempt the content word unsuccessfully (Howell, 2004a). This is a pattern seen only in the speakers who persist in stuttering and is a sign to look out for as an indication of persistence.

#### 5.2.2 Social and environmental variables

Psychological states may continue after individuals recover from a disorder (as occurs, for instance in post traumatic stress disorder), but in other cases the states disappear once the person has recovered. As well as asking whether factors are associated with the disorder at the time at which its likely persistence is more or less fully determined (age 12 plus), the question can also be asked whether those factors occur selectively in those for whom the problem persists: clinicians would then know that treating stuttering is likely to remove associated negative psychological states. The follow-up data on persistent and recovered speakers we have collected offers a unique resource to establish whether the states are epiphenomena of stuttering. To date, results have only been reported for anxiety (whilst results for temperament, self esteem and personality will be reported in the future).

##### 5.2.2.1 Anxiety

Does anxiety stay when stuttering persists, but disappear when the problem resolves? Davis, Shisca, and Howell (in press) examined Cattell and Scheier's (1961) state and trait anxiety in a subset of the 76 children known to have been stuttering as young children and who were known to persist or recover at adolescence. A group of age- and IQ-matched fluent control children were included as well. Trait anxiety refers to permanent individual differences in the response to anticipation of threatening situations. There were no differences between persistent stutterers, recovered stutterers and the controls with regard to trait anxiety. State anxiety is an unpleasant emotional arousal in the face of demanding or dangerous situations (Lazarus, 1991). The persistent speakers had higher state anxiety than controls and recovered speakers for three out of the four speaking states examined (the recovered speakers showed no state differences relative to controls). The findings were interpreted as showing that increased anxiety levels in particular states are a result of the speaking problem. This is consistent with the view that state anxiety is an effect, not a cause, of the stuttering.

##### 5.2.2.2 Other personality variables

Work on other personality dimensions also needs to compare recovered and persistent speakers. However, it is unlikely that personality factors will prove to be significant with regards to recovery, as studies have failed to find differences when persistent speakers have been compared with fluent ones. Thus, Manning, Dailey, and Wallace (1984) found that self-perceived personality characteristics of older persons who stutter (52–82 years) were not significantly different from those of nonstuttering controls. Also, Guitar (1976) showed that neither neuroticism nor extraversion, as measured by the Eysenck Personality Inventory, were predictors of recovery or persistence. One recent study has reported that adults who stutter differ from controls in psychosocial emotional scores on the Minnesota Multiphasic Personality Inventory (Treon, Dempster, & Blaesing, 2006), which might merit further investigation.

##### 5.2.2.3 Summary on socio-environmental factors on speakers at 12 plus

In summary, state anxiety differs between speakers who persist in their stuttering at teenage compared with fluent controls. When speakers recover, the state differences disappear. This suggests that treating the stutter is vital, as the associated psychological problems will then diminish. When speakers recover, their anxiety level converges to that of fluent speakers. Most studies in this age range have examined children who persist, and this has provided useful information about how the disorder develops. More needs to be known about how the problem resolves in recovered cases. Temperament, self esteem and personality remain to be investigated with regards to what happens after recovery.

#### 5.2.3 Host factors at 12 plus

##### 5.2.3.1 Performance

###### 5.2.3.1.1 Sensory

In the earlier age range, speakers who stutter had a high incidence of OME and there was some ambivalent evidence for a central auditory deficit. When recovered and persistent speakers were examined at age 12 plus the effects that were seen had implications for recovery and possibly even for prognosis. Recall that there was a high reported incidence of OME at the earlier age and a question is whether this was sufficiently acute to merit an operation? The proportion of children who had ventilation tubes fitted was ascertained and it was examined whether this was associated with persistence and recovery. Fifty percent (50%) of cases (7/14) overall were treated by ventilation tubes. Interestingly, the rate for the operation was lower for the persistent speakers at 33.3% (2/6) than recovered speakers at 62.5% (5/8).

Whether the number of cases where ventilation tubes were fitted was higher than chance can be established using data from the Doctors' Independent Network (DIN), assuming all children will have visited their general practitioner before they are five (De Wilde et al., 2004). Rovers, Schilder, Zielhuis, and Rosenfield (2004) report that OME is rare after five years of age. The DIN database showed 6.6% of children in general were referred with OME and 21% of these were treated by ventilation tubes up to age five years. Thus 1.4% (21% of 6.6%) of all children up to five years were fitted with ventilation tubes. These figures are much lower than shown by either the persistent or recovered speakers.

Fitting ventilation tubes may facilitate recovery, given that fewer children who persist (33.3%) had ventilation tubes than those who recovered (62.5%). On the other hand, the mechanism by which treating OME could improve speech control is unclear and the result needs replicating with larger samples. An alternative explanation could be parental concern (as noted in connection with comorbidity). If, however, the OME is real and more prevalent in this group of speakers, it would lead to central hearing deficits (Stephenson & Haggard, 1992). These could, in turn, be behind the reported backward masking deficits (Howell et al., 2000).

The latter argument may seem a little premature as there has been one study reporting a backward masking deficit and one that does not. However, a third study on backward masking looked separately at persistent and recovered speakers (Howell, Davis, and Williams, 2006) and appears to resolve the discrepancy between the two earlier studies. Howell et al. (2006) reported that a subset of the children who persist in their stuttering showed poorer backward masking performance than recovered speakers who were definitely known to have been stuttering at an early age. It appears that speakers who persist in stuttering may have a slight central auditory deficit (about 10 dB worse thresholds than recovered speakers), which affects processing of backward masking stimuli.

###### 5.2.3.1.2 Motor

Many studies suggest that stuttering is tied up with speech motor execution rate. Early studies examined gross rate changes such as change in mean speech rate for an entire utterance. While the majority of the studies showed an increase in mean utterance rate decreases fluency and a decrease in utterance rate increases fluency (see Howell, 2004a for an extensive review), the studies suffer from the fact that speakers have dynamic control over rate. Thus a speaker may speak rapidly at some points in an utterance and slowly at other points. For this reason, recent attention has shifted to variability in speech timing control (Howell et al., 1999; Howell & Sackin, 2000; Smith & Kleinow, 2000). The results show that stuttering is associated with local increases in speech rate (Howell, Au-Yeung, & Pilgrim, 1999; Howell & Sackin, 2000) and increased timing variation (Smith & Kleinow, 2000). No studies have been done to establish whether these effects occur specifically with persistent speakers. This hypothesis is worth examining as stalling can be regarded as a way of reducing speech rate. As the persistent speakers cease stalling, speech rate may increase that results in their fluency problem. Changes to speech timing have been a goal of many different forms of treatment for stuttering (again see Howell, 2004a for a review). One form of procedure that alters speech timing and increases speech fluency (delayed auditory feedback) affects structures in the cerebellum (Howell & Sackin, 2002). A question that will be returned to is whether involvement of the cerebellum under delayed auditory feedback can be confirmed in scanning studies (see 5.2.3.2.2).

To summarize, the clinician should be alert to speech motor timing problems. These are, to some extent, client dependent (everyone has a modal speech rate). However, the clinician may be able to determine whether dysfluency rate increases when speech rate increases and to determine whether dysfluency rate decreases when speech rate decreases.

##### 5.2.3.2 Biological

This section examines whether the individual's biological makeup contributes to whether he or she will persist in or recover from stuttering.

###### 5.2.3.2.1 CNS drug effects

Stuttering has been found to be associated with high dopamine levels. Antipsychotic dopamine antagonists have been found to reduce stuttering frequency (Maguire et al., 2004). Stuttering has also been found to be reduced in some cases by the use of selective serotonin reuptake inhibitors (Gordon et al., 1995), tricyclic antidepressants (Stager, Ludlow, Gordon, Cotelingam, & Rapoport, 1995) and anti-anxiety medications (Brady & Ali, 2000). All these medications affect the central nervous system and, the reported stuttering reduction suggests that they affect key sites in the brain that control stuttering.

###### 5.2.3.2.2 Scanning

**Fig. 1:**
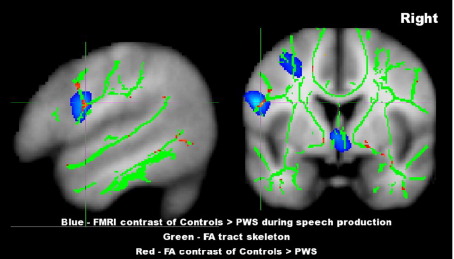
Fractional anisotropy (FA) contrasts in the white matter between controls and speakers who stutter (persistent and recovered, PWS). (For interpretation of the reference to colour in this figure legend, the reader is referred to the web version of this article.)

Scanning studies have been conducted with older speakers and show a variety of sites are active (see the issue of the Journal of Fluency Disorders mentioned previously for a comprehensive review). The studies reviewed in this journal issue, either do not look at whether persistent speakers differ from recovered speakers in any way, or rely on retrospective reports from speakers that they stuttered in the past. Our own work on the combined persistent and recovered speakers showed that, as a group, they had significantly lower fractional anisotropy in the white matter underlying ventral premotor cortex, left and right dorsal premotor cortex, and in the left and right corticobulbar tract (Watkins, Smith, Davis, & Howell, 2006) as shown in Fig. 1. These areas have been reported to be active in other studies. For instance, the lower fractional anisotropy in the left ventral premotor region is close to the white matter disconnection reported for people who stutter by Sommer, Koch, Paulus, Weiller, and Buchel (2002). Structural differences have also been found between persistent and recovered speakers in the region of the fibre bundle that connects Broca's and Wernicke's area, but further analysis is required before details can be given.

Another aspect that has been examined using scanning techniques is cerebellar activity under altered feedback in speakers who stutter. Watkins, Patel, Davis, and Howell (2005) reported significantly greater activation in a delayed auditory feedback condition relative to a normal hearing condition. The cerebellum is the region of the brain implicated earlier in timing control under delayed auditory feedback, which seems to be substantiated by these results. There appears, then, to be evidence that persistent speakers have brains that are structurally or functionally different to recovered speakers, and that fluency is affected by delayed auditory feedback by mechanisms in the cerebellum. Although, from a clinical view, these findings may have little immediate impact, they serve to further support the view that sensory and motor processes operate differently in speakers who stutter and, to some limited extent, between those who persist and those who recover from stuttering.

###### 5.2.3.2.3 Genetics

Ambrose et al. (1993) examined whether genetic transmission is different in persistent and recovered cases. They found that, in addition to the single major locus component discussed earlier, there are polygenic factors that determine the path the disorder will take (individuals with a family history of persistent stuttering also tend to persist, whereas individuals with a family history of recovered stuttering also tend to recover). Thus there appear to be specific predispositions determining the path stuttering will take. This suggests that speakers who will persist have latent genetic influences which eventually lead them to diverge from recovered cases.

##### 5.2.3.3 Summary on host factors on speakers at 12 plus

In summary, dysfluency in language samples offers a clear sign that distinguishes persistent and recovered cases. Host's performance shows a sensory deficit for backward masking stimuli (stimulus levels have to be 10 dB higher in persistent speakers than recovered speakers to be detectable). This deficit may be associated with the high rate of OME (occurs for both persistent and recovered speakers) with concomitant lower rate of ventilation tubes in persistent than recovered speakers. Though uncorrected OME may lead to hearing problems that affects fluency, the higher rate in recovered speakers may be because of higher rates of parental concern about disease in general. Motor timing processes at local levels in utterances are related to fluency, possibly involving cerebellar mechanisms. The behavioral data on sensory and motor structures in speakers who stutter are consistent with some recent scanning data.

## 6 Discussion

First the main findings are collected together. Language, social-environmental and host factors are considered in turn for the two age groups (up to age eight and 12 plus). In the language of speakers who stutter up to age eight, dysfluencies on whole function words predominate. Fluent speakers also show dysfluencies on function words predominantly, though the rate in speakers who stutter is much higher. Howell, Au-Yeung, and Sackin (1999) data show that function word dysfluencies are about four times higher in these speakers than fluent speakers in this age range. The repetitions in the extract of the five-year-old speaker given in Section 2 show this pattern insofar as they involve whole words and the words involved are function in type.

Twin studies were reviewed, which showed that environmental and host factors were split roughly 30/70 for both ages. When socio-environmental factors were examined up to age eight, there seemed to be a disparity between old studies and more recent ones. The older studies pointed to an impact of an impoverished environment on stuttering, but more recent research does not confirm this. The disappearance of these socio-environmental influences leaves the problem that there is still 30% of the environmental variability there, although it has not been possible to identify any specific such influences on stuttering. Thus, although temperament differs between speakers who stutter and controls in this age range, this factor does not seem to mediate specific language development landmarks.

When host factors were examined at these ages, there was the first suggestion of sensory (OME), and motor differences (handedness) relative to control populations. It was also noted that Alm (2005) has raised the possibility that stuttering is associated with head injury, which might mediate these performance deficits. Other biological factors show a consensus that there is a big genetic influence although there is disagreement about the mode of transmission of the disorder. No scanning evidence comparing children who stutter and controls in this age range, which could advance our understanding of these issues, has been published at present.

Language factors at 12 plus show that children who persist, diverge from the speakers who recover insofar as, for the former, the dysfluencies on content words occur at an increased rate. The dysfluencies change and involve only the first part of a word. This pattern can be seen in the dysfluencies on the content words in the extract of the 12-year-old boy given in Section 2. The dysfluencies of speakers who recover by this age reduce to the rate shown by the fluent speakers, and dysfluencies do not change in terms of which words are affected or the type of dysfluency on the words, as is seen with the speakers who persist.

In terms of socio-environmental factors at teenage, studies have started to appear that raise the question of whether, when persistence or recovery has been established for a speaker, psychological states observed between persistent and fluent speakers remain or not when a child recovers. Persistent speakers show state anxiety relative to fluent controls. Recovered speakers who were known to be stuttering at an earlier age appear to have lost state anxiety (i.e. their state anxiety is indistinguishable from that of fluent controls). Few other socio-environmental factors could be identified (as at ages up to eight). Host factors suggest that sensory performance is poorer in speakers who persist, that they have a high likelihood of having OME and that there is less chance of it being operated for compared to recovered speakers. All these suggest a sensory deficit. Scanning evidence shows some replicable abnormality in the areas connecting motor and sensory areas in speakers who stutter. These effects could be due to structural differences in these speakers or could be functional (arising because there is a past history of stuttering in these speakers). Without further evidence close to onset of the disorder, these alternatives cannot be distinguished.

These findings can be viewed in two ways: on the one hand, they raise issues about causal mechanisms of stuttering (Alm, 2005). On the other, and more pertinent for the audience of this review, they offer a set of heuristic signs about whether a child is suffering from stuttering and give some idea of how to gauge the progression of the disorder. The remainder of this review takes up this second issue and examines the ramifications of the signs that have been highlighted for diagnosis using DSM-IV-TR arranged in the four areas raised in the Introduction.

### 6.1 DSM-IV-TR diagnostic guidelines for stuttering

The first of these areas was to re-examine the recommendations DSM-IV-TR makes about how to diagnose stuttering (APA, 2000). The first point to note is that, in this reviewer's opinion, despite Wingate's arguments, DSM-IV-TR was right to include whole word repetitions as a sign for assessing the disorder. In particular, the change from whole to part-word dysfluencies occurs specifically for the persistent speakers (Table 3).

DSM-IV-TR notes that the disorder waxes and wanes in children and this makes the normal speech dysfluency that occurs in young children, difficult to distinguish from stuttering. Again there is some support for this, as presented in this review. As mentioned earlier in the discussion, fluent speakers differ from all the speakers who stutter at the early age in the rate (not in relative incidence) of dysfluencies on either whole- and part-words or function and content words. Applying a cutoff criterion based on a difference in absolute rates to distinguish fluent speakers and stutterers is difficult.

The authors of DSM-IV-TR comment that stuttering interferes with educational or occupational achievement or with social communication. While the early evidence suggested that this was the case, evidence obtained in the last 20 years or so suggests that this may not be so in contemporary society as far as educational and social communication are concerned.

DSM-IV-TR suggests that impaired social functioning may result from stuttering. Again there is partial support for this. For instance, speakers of all ages report being bullied, and this has stood the test of time. All studies done on children who stutter at an early age report that they are not anxious. The situation changes later where state anxiety is seen (relative to controls) in speakers who persist in their stuttering (but not in those who recover). These findings have been interpreted as showing that state anxiety disappears when speakers recover from the disorder. A further comment that follows on from the point about impaired social functioning is that this does not mean that all adults and adolescents who stutter have psychiatric disorders. Certainly many of the deficits associated with the disorder are within a standard deviation of the normal mean and this would not be psychiatrically significant: IQs would not qualify most stutterers as having a learning disorder. As a group the children do not have a clinically significant reading, mathematics or writing deficit, nor are they mentally retarded, their motor skill problems are too mild to meet the criteria of this disorder. The view that the deficits are mild may need qualifying insofar as they appear to be greater in persistent than recovered cases.

One omission from the current review is the usefulness of secondary symptoms in stuttering; this requires more detailed examination in the research literature.

### 6.2 Specificity of signs of stuttering

It is worth repeating that it is equally important for clinicians to decide whether a particular child is *not* stuttering as it is to determine whether the child *is* stuttering. Discussion under point one should help to fine-tune criteria used to determine whether a child is stuttering. Here disorders that can be difficult to distinguish from stuttering are examined.

Speech characteristics of other disorders described in DSM-IV-TR, show some similarity with stuttering and the question should be asked whether these differ from stuttering. To give some specific examples, is there a difference between the slurring that occurs as a result of substance abuse and prolongation (e.g. does slurring occur on the first part of content words as do stuttered prolongations)? Also, are there differences between palillalia (seen in tic disorders) and whole word repetition, possibly in the class of words repeated (stuttering involves repetition of entire function words and palillalia might involve content words). Dementia-type disorders (Alzheimer's and Parkinson's disease) show dysfluencies that are similar in some ways to stuttering. This is not crucially relevant for the current discussion because the cases concerned are from a much older population. Also, the extent of the similarity has not been properly documented.

Cases of stuttering very often meet, or almost meet, the criteria for it to be classed as another disorder. All the criteria for chronic motor or vocal tic disorder are met. Children who stutter meet all but one of the criteria for selective mutism. The exception is an exclusion criterion which requires a child not to stutter (among other things) to be classed as having selective mutism.

All except one criterion for social phobia (a type of anxiety disorder) are met. The exception is fear associated with another mental disorder where stuttering is explicitly mentioned. That is, if the fear can be put down to the stuttering, then the case does not meet the fear criterion. Again there are questions about the adequacy of an exclusion criterion in general, and in terms of the specific requirement to dissociate the fear associated with the social situation from that associated with the speech disorder per se. One possible way of doing this would be if the former was related to state anxiety and the latter to trait anxiety.

### 6.3 Comorbidity between stuttering and other disorders

Comorbidity refers to the presence of more than one disorder occurring in an individual at the same time. Overlapping diagnostic criteria would increase the chances of mistakenly finding comorbidity (area 4). One clear case of a high rate of comorbidity is that between Down Syndrome and stuttering. Preus (1972) appears to have made thorough evaluations of stuttering in children with Down Syndrome. This example raises the question whether asymmetry could be used as a way of establishing true comorbidity. As noted here, children with Down Syndrome as a group have a high rate of stuttering but, given that both are relatively rare disorders, it is doubtful that the incidence of Down Syndrome in stuttering is much higher than that of the population at large.

### 6.4 Ambiguity of classifications (risk of classifying stuttering as some other disorder and vice versa)

A second situation (apart from comorbidity) can arise when diagnostic criteria are not sufficiently precise. Cases can be misclassified (cases of stuttering designated as some other disorder, or cases of some other disorder diagnosed as stuttering). This would lead to comorbidity rates being overestimated. One mechanism that tries to reduce the chance of this is the use of exclusion criteria (discussed in Section 6.2).

There are also features of DSM-IV-TR that would work towards underreporting comorbidity. The 16 major diagnostic classes are arranged under clinical disorders (axis 1) and personality disorders and mental retardation (axis 2). Stuttering and other “Disorders usually first diagnosed during childhood or adolescence” are reported on axis 2. Axis 1 usually has precedence over axis 2, which has an impact on statements about stuttering. In particular, anxiety disorders and dementias of the Alzheimer's type are both reported on axis 1, and stutterers show many signs of the disorder. They could not be classed as having Alzheimer's and stuttering because of the axis system. While this may very well be correct, it is built into the structure of the classification scheme in a way that would reduce comorbidity. The basis for giving axis 1 precedence is not demanded by the data.

The previous point discussed issues about comorbidity across axes 1 and 2. Cross axis comorbidity does not apply to all cases where classification criteria for disorders are similar. Thus, there are also cases where both disorders are within axis 2 (e.g. stuttering and disorders like chronic motor or vocal tic disorder).

The best way of dealing with all these areas is to have more precise diagnostic signs.

## Figures and Tables

**Table 1:** Classification of 76 speakers who stutter as above or below the 50 percentile of counts of dysfluency types 4–6 per two-minute extract at the outset and end of the follow-up period

		Outset	
		Bottom 50 percentile	Top 50 percentile
End	Bottom 50 percentile	23	16
	Top 50 percentile	11	26

**Table 2:** Classification of 76 speakers who stutter at the outset of the follow-up period using the same criteria as in Table 1

		End	
		Recovered	Persistent
Outset	Bottom 50 percentile	31	11 develops
	Top 50 percentile	10 recover	24

At the end of the follow-up period the more stringent criteria for persistence and recovery (described in the text) were imposed.

**Table 3:** Average number of dysfluencies in a two-minute extract of types 2–3 on function words and 4–6 on content words at initial and final assessment for the recovered and persistent speakers

	Initial		Final	
	2–3 (stalling)	4–6 (advancing)	2–3 (stalling)	4–6 (advancing)
Recovered				
Function	3.08		1.43	
Content		1.17		0.44
Persistent				
Function	2.89		1.58	
Content		1.38		1.68
